# Validation of the Social Innovation Tendency Scale in Mexico

**DOI:** 10.3390/ejihpe15100195

**Published:** 2025-09-26

**Authors:** José Francisco Muñoz-Valle, Lucía Estrada-Pereira, Francisco Javier Turrubiates-Hernández, Alexis Missael Vizcaíno-Quirarte, Norma A. Ruvalcaba-Romero

**Affiliations:** 1 Máster en Innovación Social y Economía Solidaria, Instituto Universitario de Estudios sobre la Ciencia y la Tecnología, Universidad de Salamanca, 37008 Salamanca, Spain; 2 Centro de Innovación Social y Emprendimiento Sostenible, Coordinación de Ecosistemas para el Aprendizaje, Centro Universitario de Ciencias de la Salud, Universidad de Guadalajara, Guadalajara 44340, Mexico; francisco.turrubiates@academicos.udg.mx; 3 Instituto de Investigación en Ciencias Biomédicas, Departamento de Clínicas Médicas, División de Disciplinas Clínicas, Centro Universitario de Ciencias de la Salud, Universidad de Guadalajara, Guadalajara 44340, Mexico; 4 Licenciatura en Psicología, Centro Universitario de Ciencias de la Salud, Universidad de Guadalajara, Guadalajara 44340, Mexico; 5 Doctorado en Psicología de la Salud (Modalidad Directa), Centro Universitario de Ciencias de la Salud, Universidad de Guadalajara, Guadalajara 44340, Mexico; alexis.vizcaino@cucs.udg.mx; 6 Departamento de Psicología Básica, Centro Universitario de Ciencias de la Salud, Universidad de Guadalajara, Guadalajara 44340, Mexico; norma.ruvalcaba@academicos.udg.mx

**Keywords:** social innovation, instrument validation, factor analysis, social entrepreneurship, higher education

## Abstract

This study validates an instrument for measuring the tendency toward social innovation at the individual level within the Mexican population. The Social Innovation Tendency Scale was administered to 1129 university students in Mexico. Unlike the original unidimensional structure, the results revealed two distinct dimensions: behavioral elements (concrete actions for social change) and attitudinal elements (beliefs and values that drive social innovation). The revised structure demonstrated strong psychometric properties and acceptable fit indices, supporting its suitability for application in the Mexican population. Evidence of convergent validity and invariance by sex and semester further supports its robustness. This study contributes to a reliable instrument for assessing social innovation in higher education, offering evidence that can inform the integration of these competencies into professional training for sustainable development.

## 1. Introduction

Over time, evolutionary economics and the innovation systems approach broadened the concept of innovation beyond its technological and business dimensions, incorporating a sociological perspective. From this standpoint, innovation is an interactive process among various stakeholders, fostering the creation of new knowledge through social action (Köhler & González Begega, 2014). This perspective emphasizes the need for practical solutions to address social and economic challenges, ultimately striving to make a tangible impact on people’s lives (Goldenberg & Canadian Policy Research Networks Work Network, 2004).

Building on this perspective, the recognition of innovation as a process of social action highlights that it extends beyond the technological and business domains, being deeply intertwined with the characteristics and dynamics of society (Fernández-Esquinas, 2020). In recent years, the concept of social innovation has gained prominence as a distinct category of innovation (Marques et al., 2018). Unlike technological and economic innovations, whose primary focus is often on market competitiveness and financial gain, social innovation aims to drive social change by addressing collective needs, with economic profit not being a primary objective (Pastor Pérez & Balbinot, 2021).

The conceptual development of social innovation emerged between 2002 and 2010, giving rise to two major research strands that continue to shape the field. The first focuses on identifying social needs across multiple dimensions—environmental, economic, social, and cultural—and examining how innovation can effectively address them. The second emphasizes the transformation of networks and the role of collaboration as key strategies for achieving shared goals (Maestre et al., 2021). Definitions of social innovation may vary; however, in broad terms, it refers to innovative activities and services designed to address social needs (Foroudi et al., 2021). Moreover, a universally accepted definition has yet to be established, as the concept is complex and rooted in interdisciplinary contributions that evolve in response to distinct historical and social contexts (Satalkina & Steiner, 2022).

In Latin America, the term “social innovation” gained popularity in 2013. However, specialized studies on the topic only began to emerge after 2016, exploring the relationship between innovation and key aspects such as social responsibility, sustainability, competitiveness, participation, and community empowerment. Within this framework, growing attention has been given to social innovation ecosystems, the resilience of social innovators, and public policies that foster social innovation and entrepreneurship (Jaillier-Castrillón et al., 2020). In the face of global challenges such as poverty, unemployment, and environmental degradation, social innovation has become a key driver in the search for inclusive and equitable solutions (Martínez-Celorrio, 2017). In this context, society has taken on an active role in the implementation of social innovation strategies, fostering collaborative initiatives to address contemporary social challenges (Moreno & Calderón, 2012).

Higher education institutions (HEIs) play a crucial role in economic and social development, not only by contributing to the formation of specialized human capital, but also by consolidating values and promoting innovation with social impact (Orellana-Navarrete et al., 2022). Through social innovation projects, students apply their knowledge to real-world problem-solving within their communities, which strengthens both disciplinary and transversal skills (Gleason Rodríguez et al., 2022). As part of this transformation, several Latin America universities have created specialized institutes and programs in social innovation, aiming to implement strategies that address social challenges and foster sustainable development (Buckland & Murillo, 2014).

In this regard, developing coherent strategies to foster a stronger culture of social innovation requires starting with accurate diagnosis, achieved through the measurement of social innovation trends (Hernández-Ascanio et al., 2021). However, most existing instruments have been designed either to evaluate an organization’s capacity for social innovation (Hernández-Ascanio et al., 2021) or to assess the impact of social innovation within business and organizational projects (Buckland & Murillo, 2014). There remains a shortage of instruments that measure individual willingness to take action, develop solutions, and contribute to improving quality of life.

The literature highlights several key characteristics of individuals with a tendency toward innovation or social entrepreneurship, including social concern, a propensity for change, collaboration, risk-taking ability, decision-making skills, and continuous learning (García-Flores & Palma Martos, 2019; Patiño Castro et al., 2016). Additional attributes such as leadership, commitment to social causes, persistence, creativity, and autonomy have also been emphasized (Alvarez De Mon et al., 2021; Ferrer-Cerveró et al., 2014).

Instruments to measure these tendencies are still scarce in Latin America. Montufar Melo (2020) proposed a 30-item scale to assess entrepreneurial attitudes, while (Pasikowski & Zajda, 2018) developed an inventory in Poland to evaluate attitudes of individuals engaged in addressing local social issues. Particularly relevant is the scale by Bulut et al. (2013) tested with Turkish university students, which resulted in an eight-item unidimensional scale to assess social innovation tendency. Notably, the present study takes the original version as a reference for conducting factorial analyses.

This study is based on theoretical perspectives that define social innovation as a process of collective transformation, accompanied by a predisposition at the individual level to generate social value through social cohesion. In his early work, Schumpeter (1934) defined innovation as the introduction of novel combinations, such as markets, organizational structures, processes, or products, focusing on how these combinations could revolutionize economic systems (Schumpeter, 1934). This foundation provided the basis for traditional definitions of social innovation, which describe the concept in terms of its capacity to transform social practices and address unmet needs (Cajaiba-Santana, 2014; Moulaert et al., 2005). In addition, other approaches relate social innovation to dynamic characteristics such as creativity, collaboration, and the development of sustainable solutions within communities and organizations (Howaldt & Schwarz, 2010).

Based on this, Bulut et al. (2013) proposed the Social Innovation Tendency Scale with the aim of operationalizing this construct at the individual level, and characterizing attitudinal elements such as beliefs, perceptions, and values related to innovation, as well as behavioral elements such as actions and practices that promote change. This dual perspective aligns with theories that recognize the interaction between cognition and action in innovation processes, thereby supporting the multidimensional design of the scale. In the context of higher education, these theoretical perspectives are particularly relevant, as universities are key spaces for fostering both the mindset and practices that enable students to act as agents of social change.

Assessing the tendency toward social innovation among university students is a valuable opportunity, especially in health science education, where these competencies are key to addressing contemporary challenges. Accordingly, this study seeks to clarify whether the Social Innovation Tendency Scale (Bulut et al., 2013) is valid and reliable in the Mexican context. To this end, we pose the following research question: Does the Social Innovation Tendency Scale demonstrate adequate validity, reliability, and factorial structure among Mexican university students?

In summary, this study analyzes the factorial structure of the Social Innovation Tendency Scale and assesses its validity and reliability in a sample of Mexican university students. By confirming a two-factor solution, it provides new evidence for measuring social innovation in higher education contexts and addresses the scarcity of validated instruments in Latin America. The article first details the methodology applied, then presents the psychometric results, and finally discusses the implications for research and practice in social innovation within higher education.

## 2. Research Methodology

### 2.1. Design

An instrumental methodological study was conducted with the aim of adapting and validating the Social Innovation Tendency Scale (Bulut et al., 2013) in the Mexican university population.

### 2.2. Participants

Sample size was determined with Epidat (v.4.2), assuming an unknown prevalence (*p* = 0.50), 95% confidence, and absolute precision of 5%. Using finite-population correction for the two target populations (N = 1859 students enrolled in the first semester and N = 1382 students enrolled in the final semesters), the required minimum sample sizes were n = 319 and n = 301, respectively.

Finally, the sample comprised 1129 undergraduate students (n = 546 students enrolled in the first semester, n = 568 students enrolled in the final semesters, and n = 15 students did not report their semester of enrollment) from the *Centro Universitario de Ciencias de la Salud* (CUCS) at the University of Guadalajara, Mexico, with ages ranging from 18 to 40 years ($\bar{x}$ = 21.50, SD = 3.35). Of the participants, 33% were male, 66% female, and 1% preferred not to disclose their gender identity.

Regarding academic standing, 48.4% were first-semester students and 50.3% were in the final semesters of one of the following degree programs: Forensic Sciences, Dental Surgery, Physical Culture and Sports, Nursing, Nutrition, Podiatry, Psychology, and Medicine (Surgeon and Midwife). One point three percent did not report their semester.

### 2.3. Instruments

- Sociodemographic Characteristics Questionnaire—Collected data on gender, age, educational program, and semester completed.
- Social Innovation Tendency Scale (Bulut et al., 2013)—The original unidimensional version consists of eight items; however, for validation purposes, the full 24-item scale was used. Participants responded using a 5-point Likert scale ranging from strongly disagree (1) to strongly agree (5).
- Questionnaire to Measure Social Entrepreneurship (Capella Peris et al., 2016), adapted for Mexico by Muñoz-Valle et al. (in preparation)—A 23-item instrument using a 5-point Likert scale, designed to assess three dimensions of social entrepreneurship: innovative traits, execution traits, and social traits. The adapted version demonstrates a reliability index of α = 0.865.

### 2.4. Procedure

The translation and adaptation of the Social Innovation Tendency Scale (Bulut et al., 2013) was carried out to ensure linguistic accuracy and cultural relevance. First, two bilingual translators independently translated the original English version into Spanish. Subsequently, both versions were reconciled into a single version under the coordination of A.M.V.-Q., who serves as Head of the Translation and Editorial Support Unit at CUCS, with the support of his staff, who have extensive experience in academic translation. This version was back-translated into English to verify its consistency with the original instrument. Finally, the adapted version was reviewed and approved by the research team for application.

The instrument was administered using a convenience sampling strategy. Participants were invited to the computer laboratory, where they completed a Google Forms questionnaire that included informed consent, sociodemographic questions, and the instrument items. The responses were subsequently entered into a predesigned database for analysis.

### 2.5. Data Analysis

The captured data were processed and analyzed using the statistical programs SPSS (v.25) and JASP (v.0.19.0.0). To ensure robustness of the validation process, the total sample was randomly divided into two groups to perform exploratory factor analysis (EFA) and confirmatory factor analysis (CFA). Prior to factor extraction, the adequacy of the data was verified using the Kaiser-Meyer-Olkin (KMO) index and Bartlett’s sphericity test. For the EFA, principal component extraction with Varimax rotation was applied, considering factor loadings ≥0.40 as acceptable. Items showing low communalities or cross-loadings on multiple factors were removed. The factorial structure identified in the EFA was subsequently tested through CFA using maximum likelihood estimation. In the CFA, model fit was evaluated considering: χ^2^, IFI, TLI, CFI, and RMSEA. Items were retained or removed according to their impact on the overall fit and theoretical consistency. Reliability was determined using Cronbach’s alpha coefficient and McDonald’s omega. In addition, convergent validity was examined through correlation analyses with an external instrument (Questionnaire to Measure Social Entrepreneurship) and by computing the Average Variance Extracted (AVE). Finally, multigroup CFA was conducted to test the invariance across sex and semester (configural, metric, scalar, and residual levels) (Chen, 2007).

## 3. Results

Initially, the sample (n = 566) was randomly divided to conduct the first phase of the EFA. The KMO measure of sampling adequacy was 0.904, and Barlett’s test of sphericity was significant (*p* < 0.001), confirming that the data were suitable for factor analysis. Data were extracted using the principal components method with Varimax rotation. This analysis produced a three-component matrix, in which three items (1, 23, and 24) exhibited low factor loadings, while three other items (5, 17, and 22) loaded onto multiple components. Consequently, these items were removed, and the analysis was re-run, increasing the explained variance to 46%. These steps were conducted to refine the factorial structure and ensure that only items with strong and consistent loadings were retained. This process led to a clearer factorial solution and allowed for the identification of factors aligned with the theoretical construct.

The revised matrix, presented in Table 1, identified three factors:

1. Behavioral elements—Specific actions that contribute to social innovation (items 2, 3, 8, 9, 11, 13, 15, 16, and 20).
2. Attitudinal elements—Beliefs and aspirations related to social innovation (items 4, 6, 7, 10, and 12).
3. Social concern elements—Interest in and sensitivity to social problems (items 14, 18, 19, and 21).

However, the third dimension lacked sufficient reliability (α = 0.648). In line with the procedure followed in the original study, it is recommended that this factor not be considered. Therefore, we proceeded with a two-factor solution, consistent with theoretical expectations and statistical adequacy, which grouped the items into behavioral and attitudinal elements.

**Figure 1:**
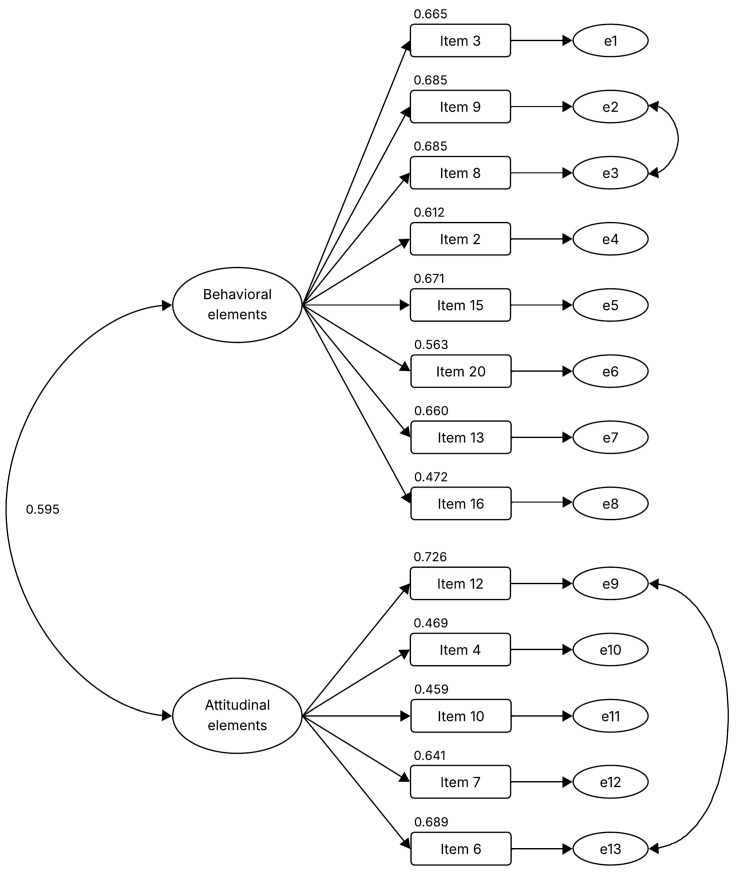
Confirmatory factor analysis with the items from the original version of the Social Innovation Tendency Scale.

As the next step, a confirmatory factor analysis (CFA) was conducted using the second half of the sample, employing the maximum likelihood estimation method, adjusted for saturated and independent models. While the initial model yielded adequate fit indices, further improvements were identified. Specifically, Item 11 was found to negatively impact the model’s indicators, leading to its removal before reanalyzing the data (Figure 1). This decision was made to obtain a refined model with improved fit while ensuring conceptual consistency.

**Figure 2:**
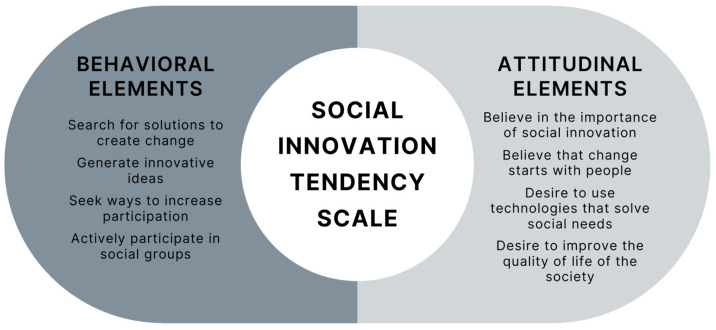
Conceptual diagram of the two-factor structure (behavioral and attitudinal elements) of the validated Social Innovation Tendency Scale.

The revised model demonstrated better fit indices: χ^2^ = 168.859, *p* < 0.001, IFI = 0.949, TLI = 0.935, CFI = 0.949, RMSEA = 0.055. The original validated scale is presented in Spanish in Appendix A. To facilitate understanding of the final factorial solution, Figure 2 illustrates a conceptual diagram of the two-factor structure of the Social Innovation Tendency Scale, showing behavioral and attitudinal elements along with representative examples of items from each dimension.

Moreover, to validate the scale with convergent instruments, a correlation analysis was conducted using the adapted version of the Questionnaire to Measure Social Entrepreneurship (Muñoz et al., in preparation). The results showed positive correlation indices, particularly between behavioral elements and innovation traits (Table 2). Additionally, convergent validity was examined through the Average Variance Extracted (AVE), Cronbach’s alpha, and McDonald’s omega. The behavioral factor obtained AVE = 0.389, α = 0.834, and ω = 0.826, while the attitudinal factor obtained AVE = 0.359, α = 0.718, and ω = 0.748.

In the invariance analysis by sex (Table 3), the unrestricted model showed acceptable indices for configural invariance. Likewise, in the analysis of progressively constrained models, the changes in RMSEA (≤0.002), SRMR (≤0.010), and CFI (≤0.006) remained within the established parameters for metric, scalar, and residual equivalence. Therefore, the model demonstrates invariance with respect to the sex of the participants. Similar results were obtained in the invariance analysis by semester (Table 3).

## 4. Discussion

The validation of the Social Innovation Tendency Scale (Bulut et al., 2013) in the Mexican population revealed a factor structure distinct from the one originally proposed by the authors. While the original study conceptualized the tendency toward social innovation as a unidimensional construct consisting of eight items, the Mexican context demonstrated a two-dimensional structure, comprising behavioral elements and attitudinal elements.

The first identified dimension encompasses the behavioral aspects of social innovation, referring to concrete actions aimed at driving social change. This category includes items related to seeking solutions for transformation, generating novel ideas, promoting social participation, actively engaging in social groups, and striving to improve living conditions.

These findings align with the existing literature on the characteristics of individuals with a tendency toward social innovation and social entrepreneurship. According to García-Flores and Palma Martos (2019), propensity for change, participation, collaboration, and the initiation of collective actions are key traits of those involved in social innovation processes (García-Flores & Palma Martos, 2019). Similarly, the ability to connect with others and a learning-oriented mindset (Alvarez De Mon et al., 2021) correspond with the behavioral dimension, where problem-solving and active participation in social groups play a crucial role.

Conversely, the second identified dimension encompasses attitudinal elements, which reflect beliefs and values associated with social innovation. This category includes items related to conviction in the importance of social innovation, the belief that change begins with individuals, the perception that technological innovation should align with social development to address societal needs, and the aspiration to improve overall quality of life.

This finding aligns with existing evidence suggesting that social innovation extends beyond concrete actions to include a psychological disposition that favors change and collective well-being. Alvarez De Mon et al. (2021) emphasize that traits such as emotional connection and a learning-oriented mindset are key characteristics of individuals with a greater tendency toward social innovation (Alvarez De Mon et al., 2021). The factorial structure observed in the Mexican population suggests that these attitudinal elements may form an independent factor, influencing an individual’s predisposition to engage in social innovation initiatives.

The differentiation of the social innovation tendency construct into two distinct dimensions in Mexico also contrasts with existing instruments. For instance, Montufar Melo (2020) developed a tool to assess attitudes toward entrepreneurship in Latin America, identifying factors such as propensity for entrepreneurial activities, perceived ineffectiveness, and risk propensity (Montufar Melo, 2020). While some of these factors may align with the behavioral elements identified in this study, the adaptation of Bulut et al.’s (2013) instrument to the Mexican context emphasizes not only action but also the attitudinal dimension (Bulut et al., 2013). This broader perspective may offer a more comprehensive understanding of social innovation, capturing both practical engagement and the underlying beliefs and values that drive individuals toward social change.

Similarly, in Poland, Pasikowski and Zajda (2018) developed the Attitude Inventory toward Social Innovation, which comprises three dimensions: attitude toward social change, attitude toward other participants, and attitude toward action directed at change (Pasikowski & Zajda, 2018). This framework aligns with the findings of the present study, as the attitudinal elements identified in the Mexican sample reflect a comparable orientation toward social transformation.

However, the distinction between attitudinal and behavioral elements in the Mexican validation suggests that social innovation is influenced not only by a general attitude toward change but also by an individual’s ability to carry out concrete actions. This differentiation highlights the importance of both mindset and execution in the development of social innovation processes.

The findings of this study underscore the importance of assessing both behavioral and attitudinal dimensions of social innovation, particularly within higher education institutions. Higher education plays a crucial role in preparing individuals with the competencies needed to tackle social challenges and drive sustainable development through social innovation (Orellana-Navarrete et al., 2022).

In this regard, the availability of accurate measurement tools enables the identification of students’ strengths and areas for improvement, supporting the development of educational strategies that cultivate both action-oriented engagement and an innovative mindset. Promoting programs that integrate training in social innovation, leadership, and interdisciplinary collaboration can significantly contribute to the formation of social innovation ecosystems within universities and their surrounding communities.

The results provide theoretical contributions to the field of social innovation research. First, the identification of a structure composed of two elements (behavioral and attitudinal) supports the notion that social innovation is not a unidimensional construct, but rather a multidimensional phenomenon that combines specific beliefs, attitudes, and actions. This coincides with conceptual frameworks that emphasize the complexity of social innovation as a process of transformation in both collective and individual levels (Cajaiba-Santana, 2014; Moulaert et al., 2005). Second, the validation of the Social Innovation Tendency Scale in the context of higher education in Mexico extends the cross-cultural use of the instrument originally presented by Bulut et al. (2013). This contributes to the literature by offering evidence that consolidates the theoretical foundation for understanding social innovation as a tendency that can be measured among individuals, not only at the community or organizational context (Howaldt & Schwarz, 2010).

From a practical perspective, the validated scale provides HEIs with a reliable instrument for examining students’ inclination toward social innovation. This instrument can be used to determine innovative profiles and guide the creation of curricular and extracurricular programs that foster skills related to social innovation. Likewise, the scale makes it possible to establish comparisons between institutions, which promotes the development of policies and strategies based on empirical data that seek to promote innovation in different educational contexts. This is particularly important in Latin America, where instruments for measuring individual tendency toward social innovation are still lacking and where universities are increasingly required to collaborate with social change (Cajaiba-Santana, 2014; Moulaert et al., 2005).

Although the validation of Bulut et al.’s (2013) instrument in the Mexican population has provided valuable information on the structure of the trend toward social innovation, it is important to recognize certain limitations. The present study was conducted exclusively among health science students from a single HEI, and the sample was selected through a convenience strategy. Consequently, the participants’ responses may reflect not only a disciplinary bias toward social action and improving quality of life, but also institutional and contextual influences that limit the representativeness of the sample and the generalizability of the findings. Moreover, cultural characteristics specific to the Mexican university context could have shaped the way in which students perceive and respond to the construct of social innovation. Therefore, it is suggested that further research extend the validation of the instrument to students in other disciplinary areas such as engineering, social sciences, and humanities, to assess whether the factor structure remains stable across disciplines. It is also advisable to replicate its validation in different regions of Mexico and Latin America, which may provide more robust results on the cross-cultural validity of the instrument.

## 5. Conclusions

In conclusion, the confirmatory factor analysis conducted in the Mexican population revealed that the Social Innovation Tendency Scale comprises two distinct dimensions—behavioral and attitudinal—contrasting with the unidimensional structure of the original instrument. These findings highlight the importance of a comprehensive approach to the study of social innovation, integrating both actions and beliefs as key drivers of change.

The results also underscore the role of HEIs in fostering these competencies among students. The validation of this instrument provides a reliable tool for assessing the tendency for social innovation in the Mexican population, enabling the identification of innovative profiles and offering both theoretical and practical implications for understanding and promoting this construct.

Future applications should extend to other academic disciplines and longitudinal studies are recommended to examine the evolution of these competencies and their impact on professional development. Taken together, these contributions advance the measurement of social innovation tendency in higher education and position the validated scale as a useful resource for guiding research, institutional practices, and policies that support social innovation.

## Figures and Tables

**Table 1:** Exploratory factor analysis of the Social Innovation Tendency Scale.

Social Innovation Trend Items	1	2	3
3. Search for solutions to create change	0.743		
9. Generate innovative ideas	0.738		
8. Seek ways to increase participation	0.687		
2. Make efforts to improve living conditions	0.580		
15. Identify problems and solutions	0.571		
11. Have the potential to make improvements in different areas	0.516		
20. Actively participate in social groups	0.491		
13. Seek opportunities for change	0.488		
16. Seize the opportunities you have	0.413		
12. Believe in the importance of social innovation		0.708	
4. Believe that change starts with people		0.665	
10. Believe that technological innovation must coincide with social development		0.628	
7. Desire to use technologies that solve social needs		0.583	
6. Desire to improve the quality of life of the society		0.583	
21. Prefer to make social changes rather than economic ends			0.799
18. Weigh social change over individual success			0.670
14. Desire to be useful to the community without expecting financial gain			0.643
19. Prefer to discuss social problems			0.502
Variance explained	28.9	9.7	7.5
Cronbach’s alpha	0.809	0.699	0.648
McDonald’s omega	0.815	0.689	0.667

**Table 2:** Descriptive statistics and correlation analysis between the subdimensions of the Social Innovation Tendency Scale and the dimensions of the adapted Questionnaire to Measure Social Entrepreneurship.

Dimensions	$\bar{x}$	SD	Innovation Traits	Execution Traits	Social Traits
Behavioral Elements of the Social Innovation Tendency	3.48	0.587	0.573 **	0.399 **	0.423 **
Attitudinal Elements of Social Innovation Tendency	4.25	0.519	0.214 **	0.271 **	0.343 **

** *p* < 0.001.

**Table 3:** Invariance indicators by sex and semester for the Social Innovation Tendency Scale.

Invariance		RMSEA	SRMR	CFI	ΔRMSEA	ΔSRMR	ΔCFI
Sex	Configural ^a^	0.060	0.059	0.940	-	-	-
	Metric ^b^	0.059	0.069	0.936	0.001	−0.010	0.004
	Scalar ^c^	0.060	0.066	0.930	−0.001	0.003	0.006
	Residual ^d^	0.058	0.068	0.928	0.002	−0.002	0.002
Semester	Configural ^a^	0.058	0.055	0.943	-	-	-
	Metric ^b^	0.056	0.061	0.942	0.002	−0.006	0.001
	Scalar ^c^	0.056	0.062	0.938	0.000	−0.001	0.004
	Residual ^d^	0.054	0.064	0.935	0.002	−0.002	0.003

^a^ Model without restrictions comparing males and females. ^b^ Model with factor loadings constrained. ^c^ Model with intercepts constrained. ^d^ Model with residuals constrained.

## Data Availability

The data described in the manuscript are available on request from the corresponding author.

## Abbreviations

The following abbreviations are used in this manuscript:

AVE	Average Variance Extracted
CFA	Confirmatory factor analysis
ECLAC	Economic Commission for Latin America and the Caribbean
EFA	Exploratory factor analysis
HEIs	Higher education institutions
KMO	Kaiser-Meyer-Olkin
SD	Standard deviation

## Appendix A

**Table A1:** Spanish Version of the Social Innovation Tendency Scale.

Tendencia a la Innovación Social	Totalmente de Acuerdo	De Acuerdo	Ni de Acuerdo ni en Desacuerdo	En Desacuerdo	Totalmente en Desacuerdo
Busco soluciones para crear cambios políticos y sociales	5	4	3	2	1
Genero ideas novedosas que agregan valor social y hacen más efectivas a las comunidades	5	4	3	2	1
Busco formas para incrementar la participación social y la cooperación social	5	4	3	2	1
Hago esfuerzos para incrementar las condiciones de vida de las personas	5	4	3	2	1
Identifico los problemas y soluciones adecuadas a las necesidades sociales para cambiar el sistema	5	4	3	2	1
Participo activamente en agrupaciones sociales sin fines de lucro (organizaciones no gubernamentales, fundaciones, etc.)	5	4	3	2	1
Busco oportunidades que cambien las normas y reglas sociales	5	4	3	2	1
Continuamente aprovecho las oportunidades que se tienen, aun cuando los recursos son limitados	5	4	3	2	1
Creo que la innovación social es de gran importancia para la generación de economías saludables de largo plazo	5	4	3	2	1
Creo que el primer paso para un desarrollo social sustentable empieza por los cambios en la mentalidad de las personas	5	4	3	2	1
Creo que la innovación tecnológica para mejorar los estándares de vida solo es suficiente si está acompañada de desarrollo social, humano y organizacional	5	4	3	2	1
Me gustaría usar nuevas tecnologías para encontrar soluciones a los problemas y las necesidades sociales	5	4	3	2	1
Me gustaría mejorar la calidad de vida de la sociedad desarrollando nuevos productos y servicios para la comunidad	5	4	3	2	1
